# Super-resolution optical microscopy for studying membrane structure and dynamics

**DOI:** 10.1088/1361-648X/aa7185

**Published:** 2017-05-31

**Authors:** Erdinc Sezgin

**Affiliations:** cmaa71851 MRC Human Immunology Unit, Weatherall Institute of Molecular Medicine, University of Oxford, OX39DS, United Kingdom; erdinc.sezgin@rdm.ox.ac.uk

**Keywords:** super-resolution microscopy, STED, PALM, STORM, NSOM, resolution limit, cell membrane

## Abstract

Investigation of cell membrane structure and dynamics requires high spatial and temporal resolution. The spatial resolution of conventional light microscopy is limited due to the diffraction of light. However, recent developments in microscopy enabled us to access the nano-scale regime spatially, thus to elucidate the nanoscopic structures in the cellular membranes. In this review, we will explain the resolution limit, address the working principles of the most commonly used super-resolution microscopy techniques and summarise their recent applications in the biomembrane field.

## Introduction

The cell membrane is a complex structure composed mainly of lipids and proteins [1]. Interactions between these molecules shape the membrane architecture as well as its function [2–4]. For instance, domains or clusters formed due to protein–protein, protein-lipid or lipid–lipid interactions may constitute catalytic platforms for cellular activities, granting the membrane a functional heterogeneity [5]. There has been extensive effort to thoroughly decipher the underlying principles of this heterogeneity and its role in membrane bioactivity. Physicochemical basis of the lateral membrane heterogeneity has been extensively studied in model membrane systems [6, 7], however, the main challenge has been—and continues to be—the lack of appropriate methodologies that could enable us to visualise these nano-scale structures directly in the live cells [8]. To achieve this, imaging techniques with high spatial resolution are required; however, the resolution of conventional microscopy is limited to  ≈250 nm which is well above the scale of domains/clusters in the cellular membranes. As a remedy to this, imaging modules that provide higher resolution, so called super-resolution microscopy, were developed in the last two decades [9–13]. In this review, we will discuss the working principles of the commonly applied super-resolution techniques in biomembrane field, discuss their recent applications to membrane biology and how they allow us to expand our understanding of cellular membrane structure and function.

## Composition and structure of cellular membranes

Cell membranes are composed mostly of lipids and proteins. The composition of the membranous structures inside the cell varies quite drastically [14]. Each organelle has specific protein components as well as different lipidomic profile. Another prominent difference is between inner and outer leaflet of the membranes which exhibit a clear transverse asymmetry. In plasma membrane for instance, phosphatidylserine (PS) lipids are mostly present in the inner leaflet while most of the phosphatidylcholine (PC) lipids are in the outer leaflet [15]. Sterols are a crucial part of biological membranes. Cholesterol, the main mammalian sterol, comprises nearly one third of the total plasma membrane [16]. The composition of the plasma membrane is quite dynamic and changes upon cellular events such as endo- and exocytosis [17].

Our current understanding of cell membrane structure is largely influenced by ‘fluid mosaic’ model proposed by Singer and Nicholson [1]. This model proposes a lipid membrane with proteins embedded in it. Primary modification to the fluid mosaic membrane model in the last decades is the lateral heterogeneity [18]. It is now accepted that cell membrane is highly heterogeneous accommodating protein and lipid clusters which modulate the bioactivity of these components. Besides protein clusters that are formed due to the preferential interaction of their components, there are lipid-driven domains, called membrane rafts [19]. These nano-structures are formed due to the differential lipid–lipid or lipid-protein interaction energies [5], and are quite dynamic temporally and spatially. Their organisation (size, lifetime, composition etc) is modulated by several factors such as actin cytoskeleton [20, 21], temperature [22] and curvature [23]. Particularly, cortical actin dynamics underneath the plasma membrane extensively shapes the plasma membrane architecture [24]. Coupling of cortical actin to the membrane leads to membrane compartmentalisation (conceptualised as ‘picket-fence model’ [25, 26])which significantly influences the mobility and organisation of the molecules residing at the membrane [20, 27, 28].

These nano-scale structures in the membrane had managed to escape the microscopic imaging due to the resolution limit of the conventional microscopy which will be discussed in the next section.

## Resolution limit

**Figure 1.**
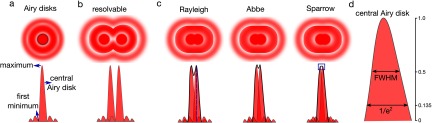
Diffraction limit. (a) When light interacts with fringes of intricate objects (such as small structures in biological samples), it broadens to a diffraction pattern as it propagates (Airy disks). This diffraction pattern becomes larger than the object masking the subtle details of it. The diffraction pattern of the light emitted by an infinitely small object forms the PSF of the object; it has a central maximum, local maxima and minima. PSF is crucial for resolution. (b) This diffraction of the light prevents the separation of two close-by objects with the standard illumination; only if the distance between two objects is larger than the resolution limit, they are seen as two separate objects. (c) If the distance between two objects is smaller than the resolution limit, they are seen as a single object instead of two separate objects. The resolution limit is defined by Rayleigh, Abbe and Sparrow differently (refer to the text for details). (d) Resolution can also be expressed by the width of the PSF such as full width at half maximum (FWHM) or the width at 1/*e*^2^.

Conventional optical microscopy techniques are limited in their resolution by a physical barrier called the diffraction limit, or Abbe limit. Resolution is the ability to distinguish closely located features, defining the minimum distance between two objects that would allow them to be visualised as two distinct objects (figure 1). In general, the spatial resolution of an optical system is approximately equal to half the wavelength of the light at which the illumination is performed. Further to this, there are a number of different approaches to more precisely define resolution which will be discussed later.

In the simplest picture, light can be considered to travel as straight rays, but on microscopic scales, light propagation is much more complex and is also governed by the laws of wave optics. When light passes a sharp edge or through a slit or opening which has a size approximately equal to the wavelength of light or smaller, diffraction patterns are generated which can only be explained by the wave theory of the light. The diffraction pattern consists of a central maximum and several higher order maxima, with the distance between each maxima depending on two parameters; the wavelength of the light and the aperture size. When light passes through a circular aperture, its interaction with the aperture forms the so-called Airy pattern (figure 1(a)) which could be used to define the resolution of the system. In optical microscopy, objective lens is used to focus the light, and it acts as the aperture to form the Airy pattern. Therefore, the properties of the objective lens generally determines the resolution of an optical setup.

Resolution limit has been defined with various different approaches. According to Rayleigh, the resolution limit is determined by:

\begin{eqnarray*}d=1.22\frac{\lambda}{f/D}\end{eqnarray*}

where $\lambda $ is the wavelength of the light, $f$ is the focal length and $D$ is the diameter of the lens. In microscopes with two different optical elements: an objective for detection and a condenser for illumination, with numerical apertures (which is the refractive index of the medium between the objective lens and the sample multiplied by the sinus of the angle at which the objective lens collects the light) $\text{N}{{\text{A}}_{\text{obj}}}$ and $\text{N}{{\text{A}}_{\text{con}}}$, respectively, $f/D$ is substituted to;

\begin{eqnarray*}d=1.22\frac{\lambda}{\text{N}{{\text{A}}_{\text{obj}}}+\text{N}{{\text{A}}_{\text{con}}}}\end{eqnarray*}

In case of fluorescence microscopy, i.e. when the objective lens also serves as a condenser, it becomes;

\begin{eqnarray*}d=0.61\frac{\lambda}{\text{N}{{\text{A}}_{\text{obj}}}}\end{eqnarray*}

This is the Rayleigh criterion for resolution; if two objects are further than this distance, they can be visualised as two separate objects (figure 1(b)), if they are closer than this distance, they cannot be resolved as two separate objects. Abbe criterion for resolution [29] is slightly different;

\begin{eqnarray*}d=0.5\frac{\lambda}{\text{N}{{\text{A}}_{\text{obj}}}}\end{eqnarray*}

While the Sparrow criterion is;

\begin{eqnarray*}d=0.47\frac{\lambda}{\text{N}{{\text{A}}_{\text{obj}}}}\end{eqnarray*}

For instance, if we use 500 nm light and an objective lens with a numerical aperture of 1.0, the minimum distance defining two ‘optically separable’ objects will be 305 nm, 250 nm and 235 nm according to the Rayleigh, Abbe and Sparrow criteria, respectively. All three metrics take into account that a higher numerical aperture and lower wavelength yield better resolution. The difference between the three resolution criteria is based on how each defines ‘optically separable’ (figure 1(c)).

Abbe calculated the resolution limit by using a grid of fine periodic structures, the illumination of which by parallel light results in a diffraction pattern in the back focal plane of the objective lens. The zero order maximum is understood as undeviated light which did not have any interaction with the specimen and therefore does not contain essential information on the sample. Thus, deviated light of at least the first order maxima is necessary to form an image. The angle at which the first order is diffracted by the sample (which is dependent on the fine structure of the grating, and thus, the dimensions to be resolved) defines the minimal aperture, which plays an essential role in resolution. The higher the NA (i.e. the larger the angle of detection), the more orders (2nd, 3rd, etc) of deviated light can be captured by the objective, resulting in better resolution. The Abbe limit applies when the two neighbouring structures cannot be resolved and instead the grid of fine periodic structures results in an image with homogenous intensity.

Rayleigh and Sparrow criteria used the size of the Airy disk (figure 1(a)) to describe the theoretical resolving power of an optical system. The 3D intensity profile of the light emitted by an infinitely small point object is called point spread function (PSF) and in a perfect optical system the PSF would be equivalent to the Airy pattern. The resolution of a microscope can numerically be expressed by applying the aforementioned criterion on the PSF. The Rayleigh criterion, for instance, is satisfied when the maximum of PSF of the first object coincides with the first minimum of the second one (figure 1(c), highlighted with the blue dashed line). On the other hand, the Sparrow criterion is met when the two PSFs are at a distance where the images no longer exhibit a dip in intensity between their maxima, but have constant intensity across this region (figure 1(c), highlighted inside the blue square). The width of the PSF is another practical measure for the resolution limit of optical systems, yet, PSF width could also be expressed differently. For instance, full width at half maximum (FWHM) is the width of the central Airy disk at the half of the intensity maximum while 1/*e*^2^ is the width of the central Airy disk at the 13.5% of the intensity (figure 1(d)).

Dependent on the maximal NAs which can be realized in modern objectives and with special immersion liquids (values up to 1.45), the best theoretical resolution for visible light is ~190 nm. Under non-perfect conditions, the resolution will be lower [30]. Nevertheless, is there any way to truly overcome this limit and resolve even finer structures? The answer is both yes and no. Diffraction as a phenomenon cannot be simply overcome, however, using fluorescence as an optical readout, the specific features of fluorescence emission, in particular the switch-like nature of fluorophores (either spontaneous or controlled switching) combined with computational post-processing, can be exploited to circumvent this limit. The possibility of selective activation and deactivation of light sources can be utilized in smart illumination and detection schemes. A completely different approach is the transition from far field to near field detection which is limited to probing surfaces by its inherently short working distances.

Although electron microscopy in principle allows shifting the wavelength to dimensions as low as single nanometers, it is not readily applicable to live cells. Therefore, many researchers have strived to overcome the barrier for optical microscopy, and several techniques have been developed during the past years, which yield access to dimensions far below the micrometer level. The most commonly used super resolution approaches will be briefly discussed below. In the perspective of biomembrane research, these techniques nurture strong hopes to directly access nano-scale organisation and dynamics of cellular membranes or other structures of functional relevance that have so far escaped optical resolution.

## Super-resolution techniques

Fluorescence is a contrasting strategy for imaging where molecules can absorb light at certain wavelengths (called excitation wavelengths) and emit at longer wavelengths (called emission wavelengths). Diffraction limit applies to both excitation and emission; the excitation light cannot be focussed on smaller spot than the diffraction limit, where emission from a point source will be expanded to a diffraction-limited PSF. Therefore, the strategies to obtain super-resolution techniques can involve modifications in both excitation and emission of the molecules.

Stimulated emission depletion (STED) microscopy, photo-activated localisation microscopy (PALM)/stochastic optical reconstruction microscopy (STORM), structural illumination microscopy (SIM) and near-field scanning optical microscopy (NSOM) are among the most commonly used super-resolution techniques. Each has certain advantages and disadvantages and usually multiple techniques are required for a complete understanding of nano-scale cellular dynamics. In the following sections, the basic principle of these techniques will be briefly introduced. We recommend the readers to refer to refs [31, 32] for recent studies comparing these techniques.

### STED

**Figure 2.**
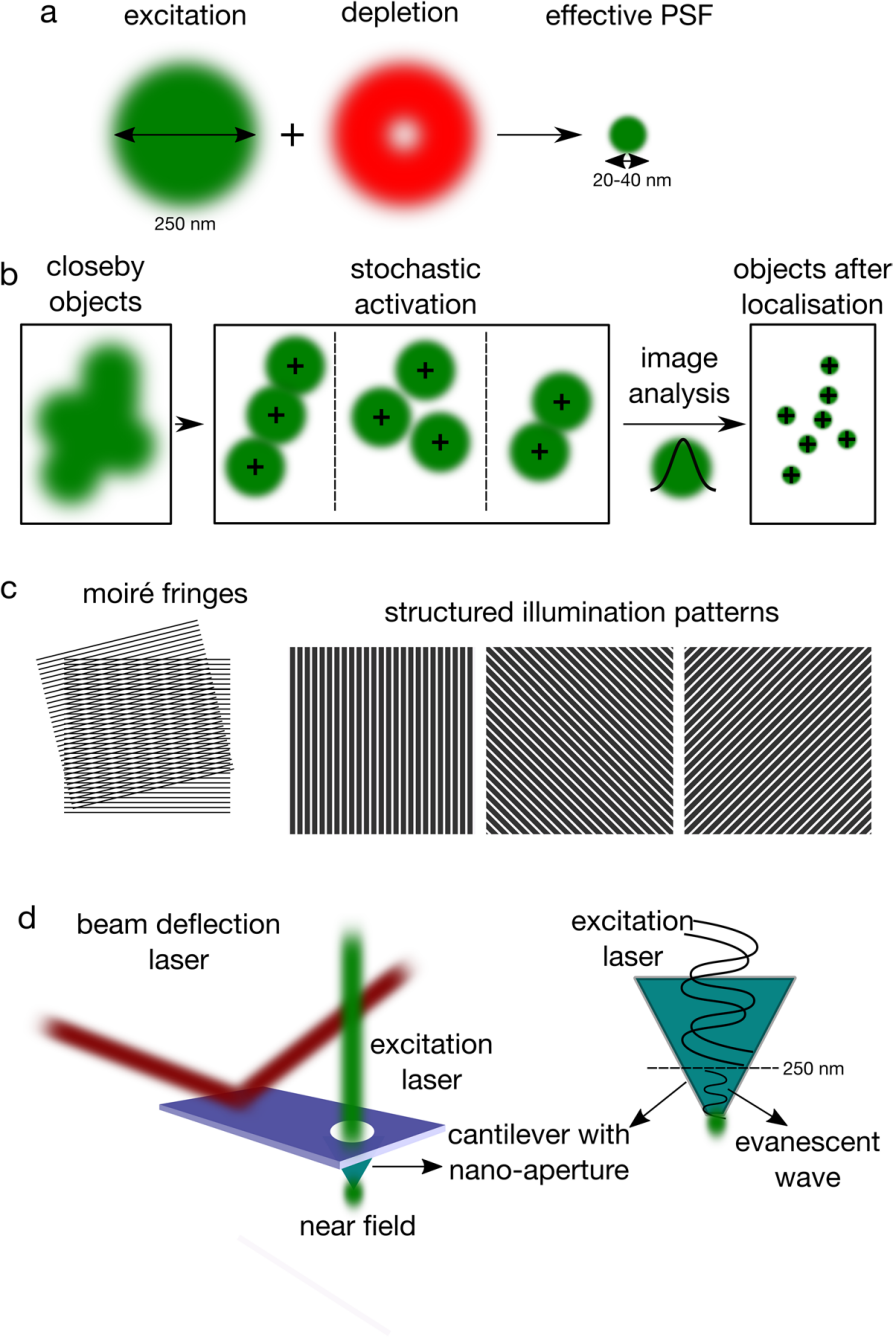
Super-resolution techniques. (a) STED is based on the depletion of PSF peripheral signal by a donut-shaped depletion beam. (b) PALM/STORM uses the photo-controllable fluorophores to observe single fluorophores at a time whose emission is then fit by a Gaussian to obtain a super-resolved image. (c) SIM is based on the predetermined illumination by a high frequency periodic light pattern, which creates interference with the high frequency variations in the fluorescence caused by small structures in the sample, resulting lower frequency Moiré interference pattern (left) which could be used to obtain information on the structures in the sample. Multiple patterns (right) are applied to obtain a single image. (d) In NSOM, size of the illumination spot is mechanically reduced by shielding of the illuminating light by an opaque screen, leaving a nanometric hole. Through this hole, an evanescent field is created which is not diffraction-limited.

The concept of STED was formulated in the 90s and experimentally realized in later years [11, 12, 33, 34]. Its underlying idea is to shape the volume which contributes to the fluorescent image of a focussed laser beam by depleting fluorophores around the immediate vicinity of the focal spot, i.e. within the disturbing periphery of the diffraction pattern. To get rid of fluorescence light in these unwanted areas, stimulated emission is performed laterally through a donut-shaped illumination by a second laser operating at a suitable wavelength (the so-called STED laser, figure 2(a)). Stimulated emission is the process of efficiently and non-destructively bringing the molecule from the excited state to the ground state without fluorescence emission, by hitting the excited state fluorophores within their fluorescence lifetime by the red-shifted STED pulse. A phase mask is used to generate the STED donut-shape profile. This allows the depletion of the peripheral signal, while keeping the STED intensity nearly zero (and thus, preserving the fluorescence) in the centre of the focal spot. The particularly neat aspect of this scheme is the nonlinear dependence of the depletion level to the STED pulse intensity; as the laser intensity increases further, the depletion region expands, but the centre of the focal spot remains largely unaffected. Therefore, the fluorescently active inner area of the PSF may be tuned continually from diffraction limited spot (≈200–250 nm, with no STED laser) down to  ≈20 nm (depending on the fluorophore), which is approximately 10 times smaller than that in confocal microscopy.

The FWHM of the effective focal spot for STED ($ \Delta r$) is described with the formula:

\begin{eqnarray*} \Delta r=\frac{\lambda}{2\text{NA}\sqrt{1+\zeta}}\end{eqnarray*}

where $\lambda $ is the wavelength of the excitation light, $\text{NA}$ is the numerical aperture of the objective and $\zeta $ is the saturation factor expressed as:

\begin{eqnarray*}\zeta =I/{{I}_{\text{s}}}\end{eqnarray*}

where $I$ is the peak intensity of the STED laser and ${{I}_{\text{s}}}$ is the saturation intensity of the fluorophore. Thus, depending on depletion laser intensity and the nature of the fluorophore, STED may offer a resolution of down to 20 nm. However, STED resolution is generally determined experimentally by using nanoscopic beads (e.g.  ≈20 nm), and calculating the FWHM (see figure 1) from the intensity profile.

STED provides an excellent resolution with relatively fast image acquisition without extensive post-processing of the images. Although this makes STED advantageous over the other super-resolution techniques for live-cell imaging, it is not yet optimal. First, the depletion laser, especially when applied at high power, bleaches most of the fluorophores that are conventionally used in cell biology, like GFP, RFP or mCherry. Another major concern is the phototoxicity caused by the high STED laser power. However, new strategies based on this imaging modality are continuously developed to minimize the laser power needed for maximum resolution. RESOLT [13], coordinate-targeted imaging with multiple off states (so called ‘protected STED’) [35] or time-gated continuous wavelength STED (cw-STED) [36] are among these improvements of the technique. STED laser can also be applied to engineer the PSF axially (called 3D-STED), yielding  ≈90 nm axial resolution [37].

### PALM/STORM

In different kinds of super-resolution approaches, PALM and STORM [38–40], the photo-activation or photo-switching cycle or spontaneous blinking property of the fluorophores is directly employed, to successively compile individual PSFs from molecules separated by more than the resolution limit, which are then deconvolved according to their centroids (figure 2(b)). Generally, all the fluorophores in the field of view is forced to enter the ‘dark state’ with light illumination. Following that, single fluorophores that are far apart (farther that the diffraction limit) are stochastically activated (‘bright state’) for each frame. Since the observation of the bright state is performed by usual excitation light that can bring the molecule back to the dark state, a blinking pattern is created by these cycles. The switching between bright and dark states is possible due to spontaneous blinking, as well. The centre of the individual PSFs are determined by a Gaussian fit. These deconvolved PSFs are plotted one by one for usually thousands of frames to finally build up the full image (figure 2(b)). This process yields images with  ≈10 nm resolution [41] depending on the localisation accuracy of the deconvolution algorithm. The resolution is now limited to the localisation accuracy, thus the resolution limit could be defined as the deviation of the spatial localisation (*σ*);

\begin{eqnarray*}\sigma _{x,y}^{~}\approx ~\sqrt{\frac{{{s}^{2}}+\frac{{{a}^{2}}}{12}}{N}+\frac{8\pi {{s}^{4}}{{b}^{2}}}{{{a}^{2}}{{N}^{2}}}}\end{eqnarray*}

Where $N$ is the number of photons; $s$, $b$ and $a$ are the standard deviation of the point spread function, the standard deviation of the mean background signal and the pixel size of the camera, respectively [42]. As the formula suggests, large number of photons and less background yield better localisation, thus better resolution.

PALM mainly employs photo-activatable fluorescent proteins while STORM requires photo-switchable or blinking fluorescent dyes (these probes will be discussed later). In the original STORM setup, the photo-switching of an organic dye is controlled efficiently when it is in close proximity with another dye (such as Cy5 and Cy3) [40]. Later, direct STORM (dSTORM) which does not require the second fluorophore was introduced [43]. Related to this, STORM requires tight control of photo-physical state of the dyes, thus specific buffer conditions are used to keep the fluorophores in the dark state until they are activated [44–46]. Despite the differences, PALM and STORM share similar working principles, so they are all together called single molecule localisation microscopy (SMLM).

PALM and STORM both yield fairly high resolution with relatively simple optical setup. They require photo-activatable/photo-switchable fluorophores as well as certain imaging media for efficient photo-activation/photo-switching, however, most of the available fluorophores can be used with these techniques and several protocols are available. The main drawback of these techniques is their time resolution. Since thousands of frames are necessary, it takes minutes to build up a complete image. Although the time resolution is improving with recent modifications, for instance on the algorithms [47], PALM and STORM are not yet optimal for live cell imaging. The temporal resolution of these techniques are below the time scale of most of the dynamics cellular processes. Also, buffers needed for STORM usually include reducing agents, and oxygen scavenger system which are toxic for the cells rendering live cell measurements challenging. Moreover, 3D imaging is quite difficult with these methods. Importantly, artefacts caused by photoswitching behaviour of the fluorophores (such as multiple activation of the same fluorophore) or labelling density should be avoided [48, 49].

### SIM

Structured illumination microscopy (SIM) takes advantage of an illumination by a high frequency periodic light pattern (usually stripes) achieved by a grating, which creates interference with the high frequency variations in the fluorescence caused by small structures in the sample, resulting lower frequency Moiré interference pattern [50] (figure 2(c)). This pattern contains sub-resolution information about the structural pattern of the sample, thus it is can be used to obtain information on the sub-resolution features in the sample by computational operations followed by illuminating the sample with various structured light patterns (obtained by for instance rotating the illumination pattern) in multiple positions [51] (figure 2(c)).

Although the resolution is rather limited compared to other super-resolution methods (≈100 nm lateral), SIM is popular as it does not need specific labelling and can work with common fluorophores unlike other techniques (i.e. fairly photostable probes for STED and photo-controllable probes for PALM/STORM). As it is a camera-based technique, it is quite sensitive and yields high contrast images over a large field of view. 3D imaging is possible with SIM (3D-SIM) as it also doubles the axial resolution (≈300 nm). The quality of the image depends on the number of different illumination patterns applied to get an image of a single plane. The more the number of these patters, the better the resolution is. Similar to PALM/STORM, its main limitation is the temporal resolution. Due to the time it takes to apply multiple patterns to get the image of a single plane, SIM is not yet ideal for live cell imaging.

### NSOM

NSOM working principle is completely different than aforementioned super-resolution techniques which are far-field imaging techniques, i.e. they use the focussed light on the sample plane. Unlike those, NSOM is a near-field technique, i.e. it is supposed to be in contact with the sample. It was first conceptualized in the 1920s [52, 53], while the first realisation took until the 70s [54]. The basic idea of NSOM is to mechanically reduce the size of the illumination light source beyond what can be achieved by optical focusing in the far-field. This basically means shielding of the illuminating light by an opaque screen, leaving a hole with dimensions in the submicron range, which is nowadays easily accessible by nano-engineering. The light emanating from this aperture, an evanescent optical near-field, is not diffraction limited (figure 2(d)). Its intensity decreases exponentially with distance, such that it is strongly confined to the surface of a sample placed in close proximity of the aperture. As discussed earlier, the resolution in far field microscopy is restricted by the wavelength of the light and the aperture of the lens. In contrast, the only factor that affects the resolution of NSOM is the aperture.

NSOM detects the evanescent field with photon detectors, thus creates a fluorescence image but also allows probing surface topology by the mechanical feedback mechanism keeping the distance between the sample and the NSOM tip constant. The combination of both optical and topological information makes NSOM an excellent super resolution technique to probe surfaces. Although NSOM gives spatially excellent resolution (a few nanometers), it operates in contact with the sample, thus the cantilever may alter the sample structure. Also, the scanning is relatively slow, making it challenging for live cell imaging.

## Probes for super-resolution imaging of biomembrane dynamics

**Figure 3.**
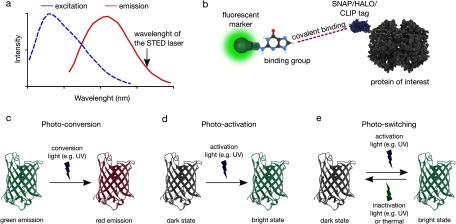
Fluorescent labelling for super-resolution techniques. (a) Spectral requirement for STED-capable dyes (b) SNAP/HALO/CLIP tag labelling strategies where a fluorescent marker that is linked to a small binding group (O^6^-benzylguanine derivatives for the SNAP-tag, O^2^-benzylcytosine derivatives for the CLIP-tag, and primary alkylhalides for the Halo-tag) can covalently be attached to the proteins that carry the SNAP, HALO or CLIP tags (c) photo-conversion is where the emission bandwidth of the fluorophore changes upon illumination with light, (d) photo-activation is triggering the bright state of the fluorophore with illumination, (e) photo-switching is reversible triggering between the dark and bright state of the fluorophore upon illumination.

STED as well as PALM/STORM techniques rely on the photophysical properties of the dyes. In STED, the first requirement is that the fluorophore should not be excited with the STED depletion laser (figure 3(a)). Since STED laser runs at fairly high powers, even a small overlap with the excitation spectrum of the fluorophore will lead to significant excitation. Second, the emission of the fluorescent probe should be non-destructively depleted by the STED depletion laser, yet it should not photobleach. While there are several dyes (such as Atto and Abberior fluorophores) performing fairly well with STED, new organic dyes are constantly being generated [55]. Fluorescent proteins (such as derivatives of yellow fluorescent protein) can also be used with STED, however, they generally do not yield high enough resolution, or they are not photostable enough. To overcome this, there is a substantial effort to develop better fluorescent proteins (brighter and more photostable) as well as novel technologies to label proteins with bright and photostable organic dyes. mGarnet [56] and E2-Crimson [57] are, for instance, two recent fluorescent proteins with which STED imaging in live cells can be performed. SNAP-, HALO-, CLIP-labeling [58–60] are amongst the protein labelling strategies where organic dyes that are linked to a small group (O^6^-benzylguanine derivatives for the SNAP-tag, O^2^-benzylcytosine derivatives for the CLIP-tag, and primary alkylhalides for the Halo-tag), can easily be attached to the proteins that carry the SNAP, HALO or CLIP tag (figure 3(b)).

For PALM/STORM, photo-activatable, photo-convertible or photo-switchable fluorophores (photo highlighters) are necessary (figures 3(c)–(e)). Emission of photo-convertible fluorescent molecules can be transformed from one fluorescence wavelength bandwidth to another (e.g. from green to red, figure 3(c)). Photo-activatable fluorescent molecules, on the other hand, are capable of being activated from a dark state to a bright fluorescent state upon usually ultraviolet illumination (figure 3(d)). Photo-switchable fluorescent molecules are reversible photo-activatable molecules, thus they can switch between a bright and a dark state upon different illuminations (figure 3(e)).

Among the commonly used photo-convertible fluorescent proteins are mEoS [61], mIriS [62] and tdEoS [63] while PA-GFP [64], PAmCherry [65] and PATagRFP [66] are the commonly used photo-activatable probes. rsEGFP2 [67] is commonly used photo-switchable fluorescent protein while there is continuous effort to produce organic photo-switchable molecules [68, 69]. More detailed review on the photo highlighters can be found elsewhere [42, 70, 71].

One particular labelling strategy worth mentioning is the nanobody technology [72]. Nanobodies are single domain antibodies, significantly smaller than full antibodies (≈15 KDa). Labelling nanobodies with organic dye of choice makes it quite appealing for super-resolution microscopy [73]. Yet, it is challenging to label the internal structures with nanobodies. A recent remedy for this is employing Streptolysin O, a bacterial toxin which forms temporary pores in the membrane and enable site-specific fluorescent labelling of proteins inside live cells [74]. Combination of these technologies will enable us to visualise the versatile structures in live cells with super-resolution microscopy.

## Super-resolution imaging of lipid/protein clusters in the plasma membrane

The plasma membrane surface accommodates different types of lipid and protein clusters [75, 76], however the functional role of the clustering on the membrane surface is still not yet fully understood. Super-resolution microscopy has been extremely helpful to expand our knowledge or sometimes even change the paradigm on both structure and function of these clusters.

**Figure 4.**
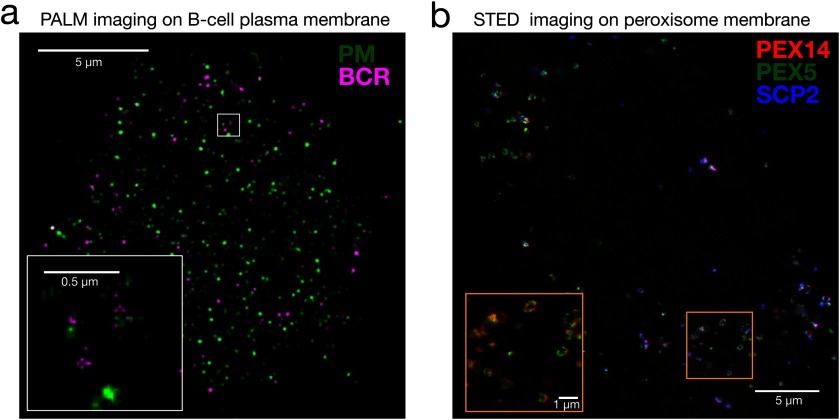
Imaging membrane structures with super-resolution microscopy. (a) Dual-colour PALM image of a B cell chemically fixed 1 min after B cell receptor (BCR) clustering. Clustered BCR is shown in magenta and a transiently expressed palmitoylated and myristoylated minimal peptide (PM) is shown in green. A weak co-localisation is observed between these two probes. Scale bars are 5 *µ*m in the large image and 0.5 *µ*m in the inset (Image courtesy of Dr Sarah Veatch, University of Michigan, Reproduced from [82]. CC BY 4.0.) (b) Dual-colour STED image of human fibroblasts fixed and immune-labelled for the peroxisome proteins PEX5 (green), PEX14 (red) and SCP2 (blue); showing the ring-like patterns. Scale bars are 5 *µ*m in the large image and 1 *µ*m in the inset (Image courtesy of Dr Silvia Galiani, University of Oxford, Reproduced from [115]. CC BY 4.0.).

STED, for instance has been applied to show the organisation of tetraspanins that form functional higher-order complexes called ‘tetraspanin-enriched microdomains’ [77]. In this study, it is shown that tetraspanin domains of different types (for instance, CD53, CD37, CD81 and CD82) do not have significant overlap, suggesting that currently established model of multiple tetraspanin species organised into a single domain may not be accurate. Organisation of immune receptors has been extensively studied with super-resolution techniques. Using PALM, it has been shown that T-cell receptor (TCR) and linker for activation of T-cell (LAT) proteins exist in separate protein islands [78]. Two-colour PALM was applied to demonstrate the organisation of LAT, TCR, Zap70, adaptor protein SLP-76 [79] and kinase Lck [80]. Recent PALM/STORM studies showed the protein re-distribution and receptor function in mast cells [81] and B-cells [82] (figure 4(a)). 3D-SIM was used to elucidate the membrane and actin reorganisation upon activation of immune cells [83, 84].

STED has been used to decipher the interaction between the plasma membrane and parasites. For instance, using STED, maturation-induced clustering of Env proteins of HIV virus that depended on the Gag-interacting Env tail has been shown [85]. Similarly PALM and STORM have been applied extensively to understand host/virus interactions [86, 87]. Super-resolution microscopy found a variety of applications in neuroscience, as well [88]. For example, STED was applied to elucidate the nicotinic acetylcholine receptor organisation [89] while PALM/STORM have been applied to decipher the nano-scale organisation of β2-adrenergic receptor clusters [90].

Lipid-driven ordered membrane domains has been a common target of super-resolution techniques. PALM/STORM studies showed the existence of sub-resolution lipid domains [91, 92] as well as domains of raft-associated proteins such as glycosylphosphatidylinositol-anchored proteins (GPI-APs)[93]. Several studies reported co-localisation of certain proteins with the raft domains [82, 94, 95]. The formation of membrane raft domains and modulation of membrane mechanical properties in relation to the underlying cortical actin structure is recently revealed using different super-resolution techniques [21]. Membrane domains were extensively studied using NSOM with artificial monolayers [96–101] as well as bilayers [102, 103]. NSOM was applied to living cells, as well where GPI-AP [104] and membrane receptor clustering were investigated [105, 106].

Studies on the clustering of the membrane components require significant attention to avoid the false positive clustering caused by technical artefacts. There has been several methodologies developed to carry out artefact-free cluster analysis [48, 49, 107].

## Super-resolution imaging of intracellular membranes and membrane trafficking

Intracellular membranous structures are excellent targets for super-resolution imaging owing to their small size [108]. Substructures in mitochondria (1 *µ*m thick), lysosomes (200–500 nm), peroxisomes (100–300 nm), endosomes (100 nm) or synaptic vesicles (50 nm) had not been imaged in detail with conventional microscopy due to the resolution limit. With the help of the super-resolution microscopy, our understanding of these structures has expanded significantly in recent years which will be exemplified below.

Bax protein is located on the mitochondrial membrane of the apoptotic cells and is one of the key players in apoptosis. Its accumulation at mitochondria and oligomerisation lead to cytochrome c release and eventually cell death. Recently, two independent studies, using different super-resolution microscopy techniques, showed that Bax organises as ring-like structures in apoptotic cells [109, 110]. The mitochondrial inner membrane organizing system (MINOS) is suggested to be the core of a protein network that controls mitochondrial function and structure. MINOS clusters and their distribution in the mitochondria has been demonstrated using STED [111]. Similarly, cytochrome c oxidase subunit 2 and voltage dependent anion channel 1 clusters in mitochondria were identified by applying STED [112].

The endoplasmic reticulum (ER) covers a large volume in the cells extending from the nuclear envelope to the cell periphery. Its contacts with other organelles serve as hubs for several processes [113]. These contacts were usually achieved by the ER sheets, however a recent 3D-SIM study showed that these sheets are instead dense tubular clusters [114].

Organisation of peroxisomes are largely unexplored as they are much smaller than most of the other organelles. Galiani *et al* recently applied multi-colour STED to show the heterogeneous spatial organisation of the peroxisomal proteins PEX5, PEX14, and PEX11, showing prominent differences between the organisation of these proteins [115] (figure 4(b)).

Membrane trafficking has been challenging to image due to the spatial and temporal scales of these processes. Although many aspects of vesicle recycling are solved, the nano-scale organisation of the individual molecules taking part in vesicle fusion/endocytosis (both on the plasma membrane and on the vesicle surface) has just started to be elucidated. It has been shown that synaptotagmin I, a vesicle membrane protein, remains clustered in isolated patches on the presynaptic membrane [116]. In the plasma membrane side, syntaxin 1 clusters define sites at which secretory granules fuse. Using STED, it has been shown that the number of clusters directly depends on the syntaxin 1 concentration as well as the SNARE motifs that ensures the homo-oligomerisation of the protein [117]. Similar clustering was observed for synaptic proteins VGluT1, synaptophysin, Rab3A and synapsin [118]. Two-colour PALM was used to shed light on the endocytic process of transferrin receptor by showing the colocalisation of transferrin with clathrin [65]. A specific limitation for tracking the complete pathway of membrane trafficking is the limited repertoire of probes. Recently, new probes have been developed to study membrane trafficking with super-resolution microscopy. Membrane-binding fluorophore-cysteine-lysine-palmitoyl group (mCLING), for instance, labels the plasma membrane and is taken up during endocytosis which makes is useful to track the endocytic pathway and to study the molecular composition of different trafficking organelles with higher resolution [119].

## Monitoring diffusion dynamics in membranes with super-resolution techniques

**Figure 5.**
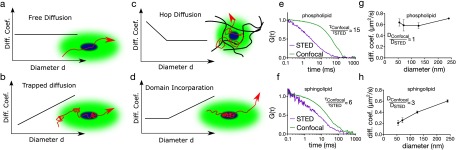
Diffusion dynamics in the plasma membrane. (a) In free diffusion, the diffusion coefficient of the molecule stays constant with varying spot diameter, (b) in transient immobilisation, the diffusion coefficient decreases with decreasing spot size, (c) in hop diffusion, the diffusion coefficient increases as observation spot gets smaller (d) in domain diffusion, the diffusion coefficient drops down but unlike transient immobilisation it levels out as the spot size gets closer to the domain size as the molecule still moves within the domain, (e) and (f) exemplary autocorrelation curves for (e) phospholipids (e.g. DPPE) and (f) sphingolipids (e.g. sphingomyelin) with confocal and STED illumination. While phospholipid transit time changes significantly (≈15 times) from confocal to STED illumination, sphingolipid diffusion time changes only marginally (≈6 times). Accordingly diffusion law of (g) the phospholipids and (h) the sphingolipids show free (where ratio of diffusion coefficient between confocal and STED is  ≈1) and transient immobilisation (where ratio of diffusion coefficient between confocal and STED is greater than 1), respectively.

Besides the structural (spatial) heterogeneity, cellular membranes exhibit a dynamic (temporal) heterogeneity. Molecules in the membrane not only diffuse with different velocities but also follow different diffusion modes [120]. The diffusion mode can simply be defined as how the diffusion coefficient of a certain molecule changes in dependence of the size of the observation spot [121]. For a molecule undergoing free (Brownian) diffusion, for example, the diffusion coefficient is not dependent on the size of the observation spot. That means the diffusion coefficient will remain constant no matter how large the observation area is (figure 5(a)). However, recent studies showed that most of the plasma membrane components do not exhibit free diffusion. Certain molecules undergo trapped (confined) diffusion (for instance due to transient immobilisation) where the diffusion coefficient drops with decreasing spot size [122, 123] (figure 5(b)), whereas most of the lipids and proteins undergo hop diffusion where the diffusion coefficient increases with smaller observation spots [27, 124, 125] (figure 5(c)) due to the cortical actin meshwork underneath the plasma membrane compartmentalising it [25]. When a molecule is confined in a domain, the diffusion coefficient only decreases to a certain point and later it stays constant [28, 120] (figure 5(d)) as the molecule freely moves inside the domain. This heterogeneous diffusion characteristics of molecules provide insight on their nano-scale spatiotemporal organisation [3]. An elucidation of the heterogeneous behaviour of membrane components is obtained by measuring the diffusion mode; the diffusion coefficient at different observation areas [121]. In a conventional microscope, the observation area is a diffraction-limited spot. By enlarging the observation spot size and tracking the molecular movement, the nano-scale dynamics of the molecules can be extrapolated from the diffraction-limited regime [126, 127]. However, with the super-resolution techniques, the observation spot diameter can be reduced and nano-scale dynamics can be studied directly. For instance, a particularly exciting application of STED to bio-membrane research is its combination with fluorescence correlation spectroscopy (FCS). FCS yields correlation curves from which diffusion time of a molecule through the focal volume can be obtained [128]. Combination of STED with FCS (STED-FCS) enables us to have access to tunable spot sizes; changing the depletion laser power allows us to tune the size of the observation spot from a confocal spot down to  ≈40 nm [123]. Diffusion mode can then directly be obtained by investigating the dependence of the diffusion coefficient to the diameter of the observation spot. This approach successfully revealed anomalous diffusion of membrane components; while phospholipids usually undergo hop or free diffusion (figures 5(e) and (g)), sphingolipids and glycolipids exhibit transient immobilisation (figures 5(f) and (h)) [122, 123, 129–131]. GPI-APs interestingly undergo domain-like diffusion [28]. Advanced modalities of STED-FCS such as gated STED-FCS [129], scanning STED-FCS [132] and STED-FLCS [133] have been quite useful to elucidate the spatio-temporal heterogeneity in the plasma membrane. Combination of STED with single particle tracking technique also showed similar anomalous behaviour of the plasma membrane components [132].

Although the limited scanning speed is the main disadvantage of NSOM when applied to membrane dynamics, it can be coupled to fast single molecule techniques like FCS [134] creating a great potential in real-time studies on lipid/protein dynamics in the plasma membrane. Similarly, PALM could be used with single particle tracking to track the movement of molecules at the cell membrane [135].

## Conclusion and future perspective

Aforementioned super-resolution techniques are the most commonly used but not the only ones. Particular biological questions may require specialised super-resolution techniques; super-resolution optical fluctuation imaging (SOFI) [136], bleaching/blinking assisted localisation microscopy (BALM) [137], super-resolution radial fluctuations (SRRF) [138], photobleaching microscopy with non-linear processing (PiMP) [139], biaxial super-resolution (BSR) [140], Cryogenic Optical Localisation in 3D (COLD) [141] are amongst these techniques. All of these techniques have particular advantages and disadvantages. An ideal microscope should have high spatial and temporal resolution and be able to image over a large field of view for a moderately long time, preferably with low illumination intensity. Although the techniques mentioned above allowed us to have access to an order of magnitude smaller scale than conventional light microscopy, they cannot meet all of these requirements simultaneously. Therefore, their optimisation as well as emergence of new techniques will continue. One such technique is SIM coupled to total internal reflection microscopy [142] (TIRF-SIM) which yields moderate lateral (100 nm) and excellent axial resolution (150 nm), however since it is based on total internal reflection, it is limited to the basal plane of the sample. Another recent technique is the MINFLUX [143], where the emitter is probed with a local intensity minimum thus reducing the photons needed for high localisation accuracy. This imaging modality gives  ≈5 nm resolution, almost the size of an antibody. This implies that as the imaging technologies progressively achieve higher resolution, imaging strategies should also catch up with smaller and brighter fluorophores. Also, elucidating the dynamic events in the live cell is preferred to capture the non-equilibrium state of the processes and to avoid the artefacts caused by fixation protocols [144]. Thus, current super-resolution methods should be improved to perform better on live cells. Meanwhile, protocols such as reversible cryo-arrest [145] will be quite useful. Eventually, combination of super-resolution techniques with advanced spectroscopic techniques such as single molecule spectroscopy [123, 132] and force spectroscopy [146] will be useful not only to image the subcellular structures but also to elucidate their dynamics.

Smart molecules such as solvatochromic probes which change their emission depending on the molecular ordering of the membranes [147, 148] are extremely useful molecules for membrane research. These dyes are used not only to visualise the membrane structures but also to quantify the physicochemical properties of the membranes such as the lipid packing [149, 150]. Combination of these probes with super-resolution microscopy is not achieved yet, thus progress in this way will also be utterly important for the membrane research.

Most of these outstanding techniques I reviewed above have been developed in the last two decades. Therefore, it is quite likely that this area will continue to be extremely exciting and in the next years, we will witness the development of many more of these imaging modalities.
